# Multi-objective optimization using improved NSGA-II for integrated process planning and scheduling problems in a machining job shop for large-size valve

**DOI:** 10.1371/journal.pone.0306024

**Published:** 2024-06-25

**Authors:** Junqiang Wang, Lihua Xu, Shuangqiu Sun, Yunfei Ma, Guofeng Yu

**Affiliations:** 1 Department of Mechanical and Electrical Engineering, Hebei Vocational University of Technology and Engineering, Xingtai, China; 2 Valve Intelligent Equipment Engineering Research Center of Hebei Province, Xingtai, China; 3 School of Future Technology, Tianjin University, Tianjin, China; 4 Yuanda Valve Group Co., Ltd., Xingtai, China; Cyprus International University Faculty of Engineering: Uluslararasi Kibris Universitesi Muhendislik Fakultesi, TURKEY

## Abstract

This paper studied an integrated process planning and scheduling problem from a machining workshop for large-size valves in a valve manufacturing plant. Large-size valves usually contain several key parts and are generally produced in small-series production. Almost all the parts need to be manufactured in the same workshop at the same time in the plant. Facilities have to handle various items in one order, including different models, sizes, and types. It is a classical NP-hard problem on a large scale. An improved NSGA-II algorithm is suggested to obtain satisfactory solutions for makespan and manufacturing costs, which involve large optimization parameters and interactions. A two-section encoding method and an inserting greedy decoding method are chosen to enable the algorithm. The dynamic population update strategy based on dynamic population update and the adaptive mutation technique depending on the population entropy changing rate are selected for enhancing both the solution quality and population diversity. The methodology was successfully implemented in a real-life case at a major valve machining workshop operated by Yuanda Valve Company in China. By taking into account realistic factors and restrictions that have been identified from a real-world manufacturing setting, this technique aids in bridging the knowledge gap between present IPPS research and practical valve production implementations.

## Section 1: Introduction

**Fig 1:**
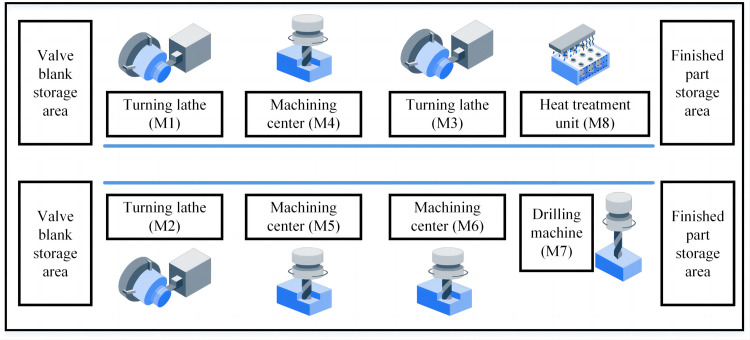
The layout illustration of the machining workshop for large-size valves.

Valves are the "throat" of the industrial pipeline system in thermal power plants, water conservancy projects, oil refineries, ports, and vessels [1]. Unlike small-size valves manufactured on the professional production line, large-size valves are produced in a single piece or small-series production. Therefore, the main machining workshops of related companies are usually built as job shops with general equipment for valve manufacturing. Fig 1 shows the machining workshop for large-size valves located within the Yuanda Valve Company of China. It is a typical layout of a job shop. The workshop contains 4 types of general machines: 3 turning lathes, 3 machining centers, 1 drilling machine, and 1 heat treatment device. 2 storage areas are set at both ends, which are the valve blank storage area and the finished part storage area. Besides, the transfer system consists of 2 overhead cranes and 4 transfer trolleys. Key parts of the large valve are machined in this workshop before assembly.

A large valve usually contains several key parts. Each part has different process plans, with several machining equipment for each process [2]. Two of the most important subsystems in manufacturing systems are process planning and machining scheduling, both of which affect the makespan [3]. The conflict between the two independent subsystems limits the machining workshop in production capacity, which is challenging even for experienced dispatchers in traditional valve manufacturers [4]. This is a classic illustration of an integrated process planning and scheduling (IPPS) problem in a manufacturing system equivalent to a job shop, which has become a topical issue in the field of production planning. Since the IPPS problem for the job shop setting is non-polynomial (NP)-hard [5], it is a challenge to find the optimal solutions in a limited time using traditional techniques. As a result, numerous scholars have put up many solutions for IPPS problems. An algorithm-based approach is a major approach [6, 7] among them [8–10].

Some popular algorithms such as agent-based ant colony optimization (ACO) [11], simulated annealing (SA) [12], and particle swarm optimization [13], have been applied to the IPPS problem. Genetic algorithm (GA) is a widely recognized heuristic algorithm in global optimization that mimics the process of natural evolution. GA was initially applied to a scheduling problem by Davis [14]. Then it has become the most popular algorithm for resolving IPPS problems. Shokouhi et al. [15] developed a GA method to maximize the makespan while minimizing the essential machine stress and the TMW. In conjunction with ACO, Zhang et al. [16] suggested an object-coding GA method and an effective meta-heuristic rule. Mohapatra et al. [17] provided a control for the privileged non-dominated sorting GA that included lowering machine idle time, processing costs, and production time. A multi-objective IPPS problem was solved by Luo et al. [18] using an efficient GA method combined with immunity principle and external archives. Amin-Naseri et al. [19] presented a hybrid GA in which the algorithm’s performance is enhanced by integrating a local search technique. An enhanced hybrid GA with variable neighborhood search (VNS) was presented by Sun et al. [20] to solve the flexible job shop scheduling problem (FJSP) while taking into account the balance of machine workload.

The Non-Dominated Sorting Genetic Algorithm (NSGA-II) is an enhanced algorithm for solving the multi-objective IPPS problem based on GA. For the scheduling problem, NSGA-II was implemented to obtain the trade-off between two conflicting objectives, maximum utilization, and minimum costs [21]. Mohapatra et al. [22] employed NSGA-II to solve the IPPS problem by considering criteria such as makespan, machining cost and machine utilization. Guo et al. [23] introduced a new NSGA-II algorithm technique to solve the proposed multi-objective integrated model for minimizing goals of maximal makespan, minimal equipment load, and least energy consumption. In a workshop on battery packaging machines, Wen et al. [24] presented a two-stage solution framework for the IPPS problem based on NSGA-II. The basic NSGA-II is employed to optimize the flexible process planning stage, and an improved NSGA-II is designed to find the non-dominated scheduling plans in the job shop scheduling stage. Liu et al. [25] employed NSGA-II to solve the proposed IPPS model and optimized the emissions of carbon and the makespan parameters. To tackle the low-carbon scheduling problem, a hybrid approach was presented by Zhang et al. [26] to handle the makespan, equipment workload, and carbon emission variables. The hybrid algorithm combines the neighborhood search algorithm and NSGA-II. For multi-objective FJSP, Deng et al. [27] introduced a bee evolutionary guiding nondominated sorting genetic algorithm II (BEG-NSGA-II) with the goals of minimizing the maximal finishing time, the workload of the machine that is most laden, and the overall workload of all machines. Wang et al. [28] minimized the mean makespan and the worst makespan across all scenarios by combining the elitist nondominated sorting NSGA-II method with tabu searching operators.

The coding and decoding methods directly reflect the design idea of the algorithm. The improvement of genetic operators is generally to develop novel encoding and decoding methods, for fitting the characteristics of the IPPS problem [29]. Liu et al. [30] proposed an integrated encoding and decoding method based on AND/OR graphs for the GA in solving the IPPS problem in the nonstandard equipment workshop for packaging machines. Fan et al. [31] created problem-specific encoding and decoding techniques to solve the flexible job shop scheduling problem. Zhang et al. [32] solved the IPPS problem using a multi-layer integrated coding approach to design genetic operators for each layer separately to accomplish iterative optimization.

In this research, an improved NSGA-II method is developed for the IPPS problem with multiple goals of minimizing the makespan, power consumption as well as cutting tool consumption. A two-section encoding method and an inserting greedy decoding method are chosen for the optimization model. The dynamic population update strategy and the adaptive mutation technique are selected for enhancing both the solution quality and population diversity. The proposed algorithm has been applied in a real-life case at a valve machining workshop. The results show that the suggested method’s efficacy is confirmed in an actual industrial scenario.

The rest of the paper is structured as follows. The IPPS problem for the machining shop is described in Section 2, and the NSGA-II framework and approach are described in Section 3 in detail. Section 4 presents a case study from a valve production plant. Sections 5 and 6 present a case study from a valve production plant and discussion. The final section concludes the paper and provides future directions for research.

## Section 2: Representations of the IPPS problem

### Machining features and processing information of the valve part

**Fig 2:**
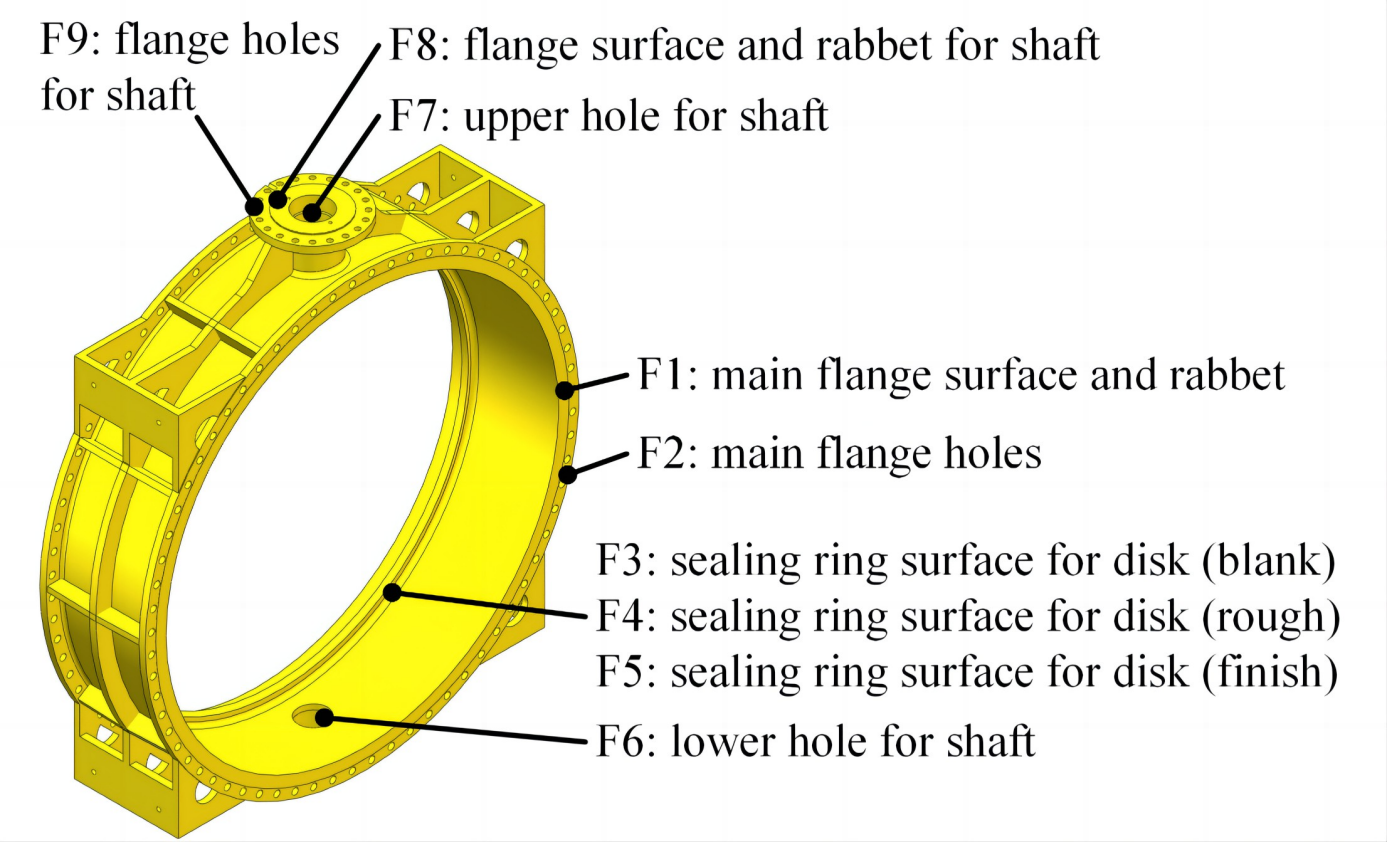
Machining features of a butterfly valve body.

Usually, a large valve contains several parts. Each valve of large size contains several machining features. All the features should be manufactured, and each feature needs at least one operation on alternative machines. In the machining workshop of Yuanda Valve Company, a butterfly valve body that consists of 9 key machining features is shown in Fig 2 as an example.

**Fig 3:**
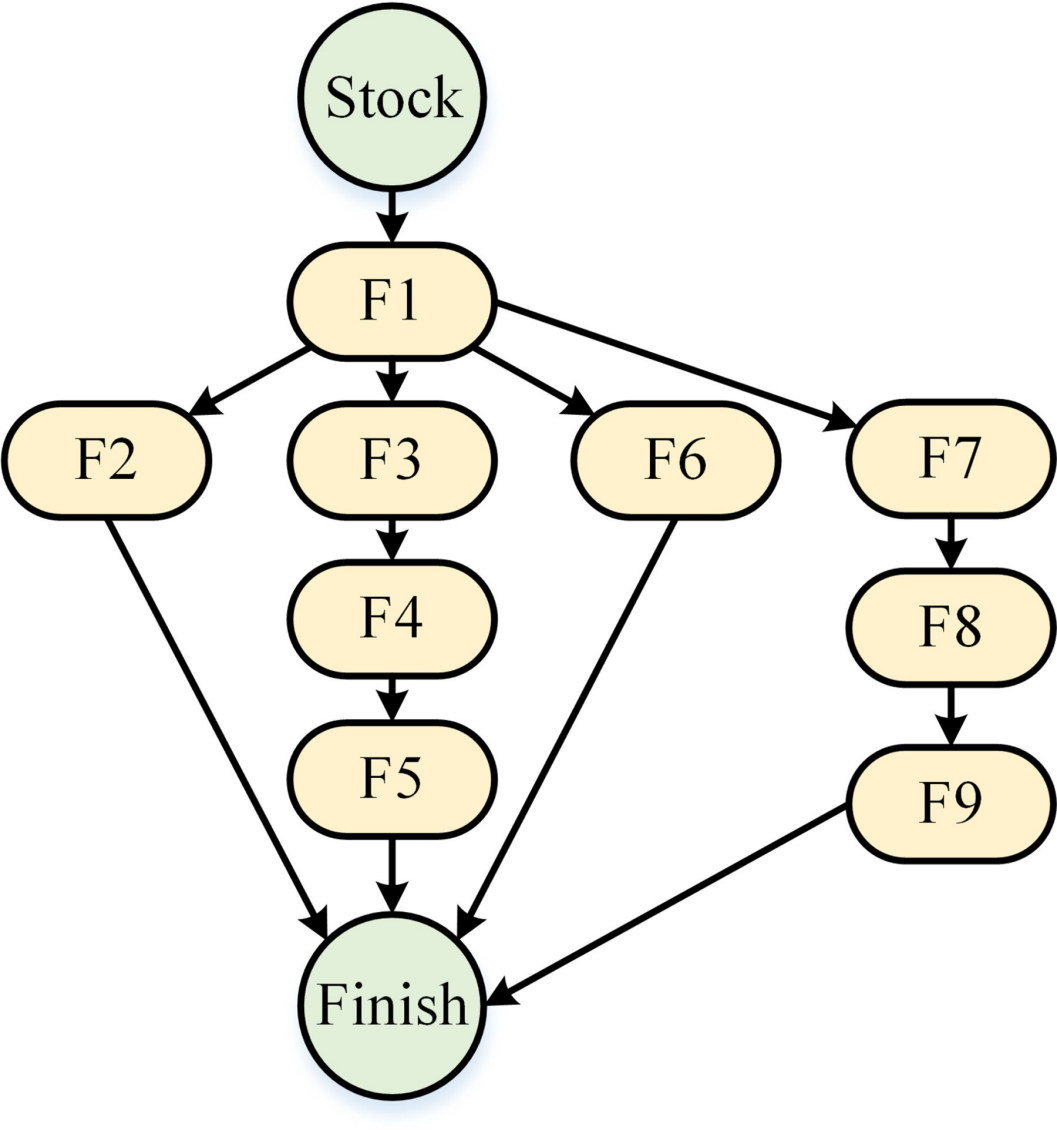
Sequence relationship network of features.

For machining features of a part in the machining workshop, the processing sequence is not fixed. Referencing the process technology for valves and the reality of the machining workshop, the sequence relationships between features of the valve body are given in Fig 3. It can be found that:

1. The “Stock” and “Finish” nodes are virtual. “Stock” represents the rough stock part, and “Finish” represents the finished part. The boxes represent the features that need to be manufactured.
2. The arrows indicate the manufacturing sequence of each feature. The feature at the beginning of the arrow should be manufactured before the feature at the end. For example, Feature 1 should be processed before Feature 2 because the feature of the main flange surface is used as a positioning reference for the feature of main flange holes according to the mature processing technology.
3. There are several branches in the network. For features in different branches, there is no sequence constraint. For example, Feature 2 can be inserted into branch “F3, F4, F5” in any position. That is, Feature 2 can be inserted into the branch before Feature 3 (processed before Feature 3), or between Feature 3 and Feature 4 (processed after Feature 3 and before Feature 4), and so on.

Processing information for the part in Figs 2 and 3 are listed in Table 1. It can be found that:

1. Alternative operations. Some features have alternative operations. For example, Feature 1 has 2 alternative operations, namely Operation 1 and Operation 2. While Operation 3 and Operation 4 are the two alternate operations for Feature 2.
2. Alternative machines. Each operation could be processed on alternative machines at different times. Operation 1 can be executed on Machine 1, Machine 2, or Machine 3 with different processing times. Operation 2 can be executed on Machine 4, Machine 5, or Machine 6 with different processing times.
3. Precedence constraint. There are precedence constraints between some processes because of feature sequence relationships. Operation 3 (or Operation 4) has to be executed after Operation 1 (or Operation 2) because holes on the flange (Feature 2) should be processed after the flange (Feature 1) is made.
4. Operation interchange: Machining sequence of some features can be interchanged if there isn’t a sequence constraint among them. Operation 3 and Operation 7 can be interchanged because there isn’t a sequence limitation between Feature 2 and Feature 4.

**Table 1:** Related processing information of the butterfly valve body.

Features	Feature description	Alternative operations	Operation description	Alternative machines	Machining times (hr)	Precedence constraints
F1	Main flange surface and rabbet	O1/O2	Turning/Milling	M1,M2,M3/M4,M5,M6	6.0,7.8,8.4/6.3,6.2,5.5	First of all
F2	Main flange holes	O3/O4	Milling/Drilling	M4,M5,M6/M7	6.3,6.0,5.4/5.1	
F3	Sealing ring surface for disk (blank)	O5/O6	Turning/Milling	M1,M2,M3/M4,M5,M6	3.0,3.6,4.2/3.5,3.0,2.7	Before F4
F4	Sealing ring surface for disk (rough)	O7	Welding	M8	4.2	Before F5
F5	Sealing ring surface for disk (finish)	O8	Turning	M1,M2,M3	6.5,7.8,9.4	
F6	Lower hole for the shaft	O9/O10	Turning/Milling	M1,M2,M3/M4,M5,M6	1.2,1.5,2.2/1.5,1.3,0.9	
F7	Upper hole for the shaft	O11	Milling	M4,M5,M6	1.5,1.2,0.9	Before F8
F8	Flange surface and rabbet for shaft	O12/O13	Turning/Milling	M1,M2,M3/M4,M5,M6	0.9,1.5,1.8/1.2,0.9,0.8	Before F9
F9	Flange holes for shaft	O14/O15	Milling/Drilling	M4,M5,M6/M7	1.2,1.5,1.8/1.5	

### IPPS problem definition

The IPPS problem can be defined as follows [33]: A job set (*J*_*1*_, *J*_*2*_, … *J*_*n*_) with *n* jobs to be processed on a machine set of *m* machines (*M*_*1*_, *M*_*2*_, … *M*_*m*_). Each job involves several manufacturing features, all of which can be handled with an array of processing methods. There could be different processing orders for the job if the limitations are satisfied. Each step of the job could be performed on an alternate machine.

**Fig 4:**
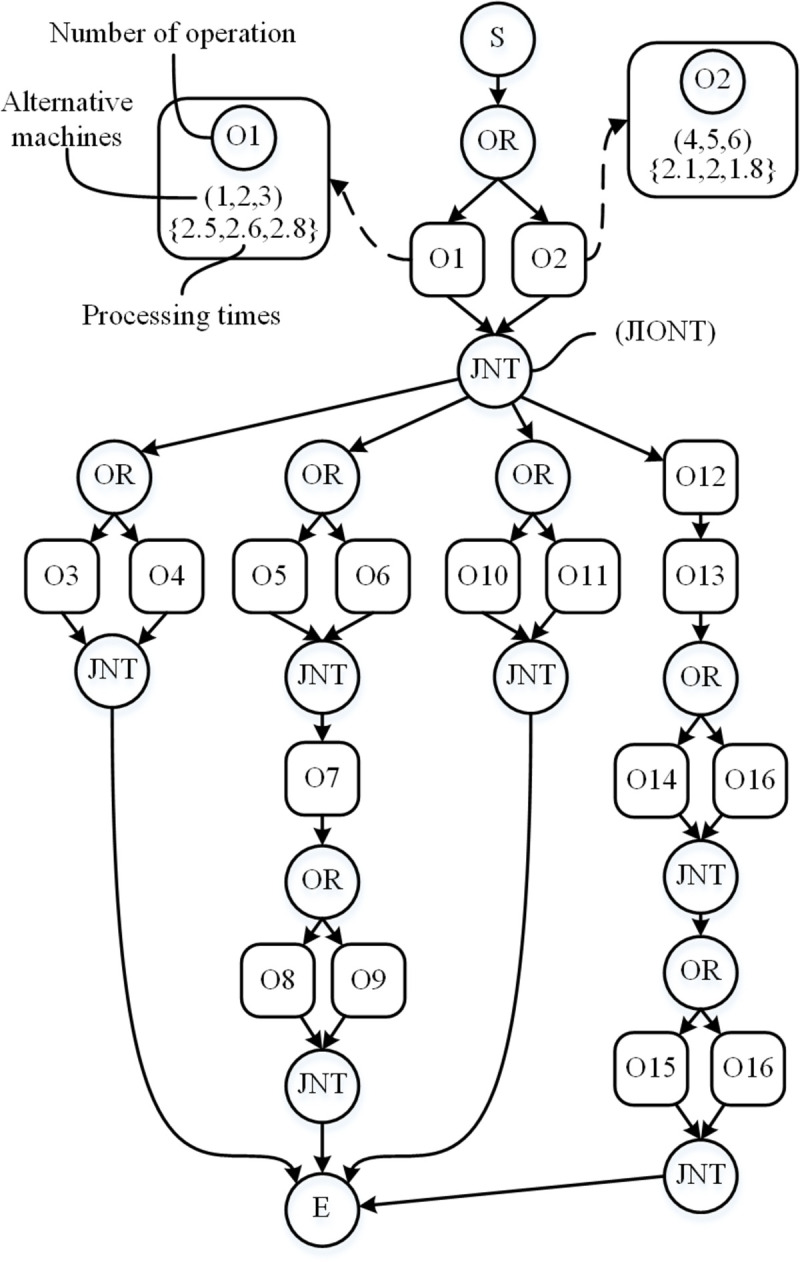
Simplified AND/OR graph of the butterfly valve body.

This problem can be expressed by a flexible process plans network graph, as shown in Fig 4. The technical specifications for the butterfly valve body presented in Table 1 are transformed into a simplified flexible process planning network. The entire collection of practical and alternate production procedures and sequences for each part are documented in an AND/OR graph [34].

In this study, three kinds of flexibility are considered: processing flexibility, sequencing flexibility, and operating flexibility. This network in Fig 4 consists of a single-directional acyclic graph with three different types of nodes and a directed edge that serves as the precedence link between the nodes. The “S” and “E” nodes are both virtual nodes. They represent the starting and ending nodes, separately. Nodes for the actual processing operations are represented by the boxes.

The processing sequence is indicated by the arrows. “OR” stands for processing flexibility, meaning that many processes can handle this feature. A job can only be processed through one “OR” if a node’s following route is connected by an “OR”. A processing path from “OR” to “JOIN” is indicated by the “OR” route. All of the operations on this line need to be visited if the path does not connect to “OR”.

For instance, a possible path for a job is: O1(M1,M2,M3)-O3(M7)-O5(M1,M2,M3)-O7(M8)-O8(M1,M2,M3)-O10(M1,M2,M3)-O12(M4,M5,M6)-O13(M4,M5,M6)-O14(M1,M2,M3)-O15(M7). The first number reflects the associated operation, and the second number indicates the chosen machine to the operation.

The practical processing operation nodes in the AND/OR graph are simplified in this paper for readability purposes. In Fig 4, a dotted arrow from the box “O1” represents the detailed content of Operation 1 listed in Table 1: operation number, alternative machines, and processing time of each machine.

### Mathematical modeling for the IPPS problem

Before solving this problem, several assumptions are constructed based on the real-world manufacturing environment for the job shop:

1. All machines are accessible at the start time. Every machine is operational at the beginning and there are no malfunctions.
2. A particular job could just be executed by a single machine at one time. Each machine can deal with a single job at one time. Once a process starts, it cannot be interrupted.
3. One job cannot have its several activities processed simultaneously.
4. There are sequential priorities between processes for a single job, whereas there isn’t any sequential priority among processes of distinct jobs.
5. Setup is part of the operation on a machine, and its time is taken into account in the processing time.
6. A job should be directly sent to the next machine following its procedure after being processed on a machine. Transmission times between machines are not computed independently; instead, they overlap with processing times.
7. Real-time stock level is less than the maximum stock capacity for the warehousing system.
8. Loading and unloading time is constant for all the jobs on all the machines.

The optimization objectives are the shortest manufacturing time and the lowest machining costs for the entire machining line. The symbols in the mathematical model are provided in Table 2. The following is a statement of the multi-objective IPPS mathematical model that this research addresses:

**Table 2:** Explanation of the mathematical model’s symbols.

Symbol	Description
*n/m*	quantity of job/machine
*M*	the current machine
*MT*	the maximum machining time among all the machines
*LT*	the total loading and unloading time for all the jobs on the machine of *MT*
*mt*_*i*_	the total machining time of Machine *i*
*mt*_*j*,*i*_	the machining time for Job *j* on Machine *i*
*lt*_*i*_	the loading and unloading time on Machine *i*
*lt*_*j*,*i*_	the loading and unloading time for Job *j* on Machine *i*
*MC*	the total machining cost of all parts
*TC*	the total tool cost of all parts
*MTC*	the total transmission cost among machines
*mc*_*i*_	the manufacturing cost of job *i*
*tc*_*i*_	the tool cost of job *i*
*mtc*_*i*_	the cost per conversion for Machine *i*
*α*	the conversion factor of two working machines

*f*_1_: Minimum manufacturing time and costs for the entire machining line.


$$\mathrm{Min}\;f_{1}=\min\;f_{2}+\min\;f_{3}$$ (1)

*f*_2_: The minimum maximal completion time (Makespan).


$$\mathrm{Min}\;f_{2}=\min\;T=\min(MT+LT)=\min(\max(mt_{i}+lt_{i})),\;i\in [1,m]$$ (2)

$$MT_{i}=\sum_{j=1}^{n}mt_{j,i},\;i\in [1,m],\;j\in [1,n]$$ (3)

$$LT_{i}=\sum_{j=1}^{n}lt_{j,i}\times \alpha (m_{i},m_{k}),\;i,k\in [1,m],j\in [1,n]$$ (4)

*f*_3_: The minimum manufacturing costs of all the machines.


$$\mathrm{Min}\;f_{3}=\min C=\min(MC+TC+MTC)=\min(\sum_{i=1}^{n}mc_{i}+\sum_{i=1}^{n}tc_{i}+\sum_{i=1}^{n-1}\alpha (M_{i},M_{j})\times mtc_{i})$$ (5)

Note that, if two adjacent processes are executed on different machines, the loading and unloading time needs to be calculated. If they are carried out on the same machine, there is no need to calculate the loading and unloading time. Hence, *α* is calculated according to the following equation:

$$\alpha (M_{i},M_{j})=\left\{\begin{array}{cc} 1 & if\;M_{i}\neq M_{j}\\ 0 & if\;M_{i}=M_{j} \end{array}\right.$$ (6)

According to the actual situation of Yuanda Valve Company, tool change time and cost are ignored because of the automatic tool-changing method.

## Section 3: Improved NSGA-II algorithm

### Basic iteration framework

**Fig 5:**
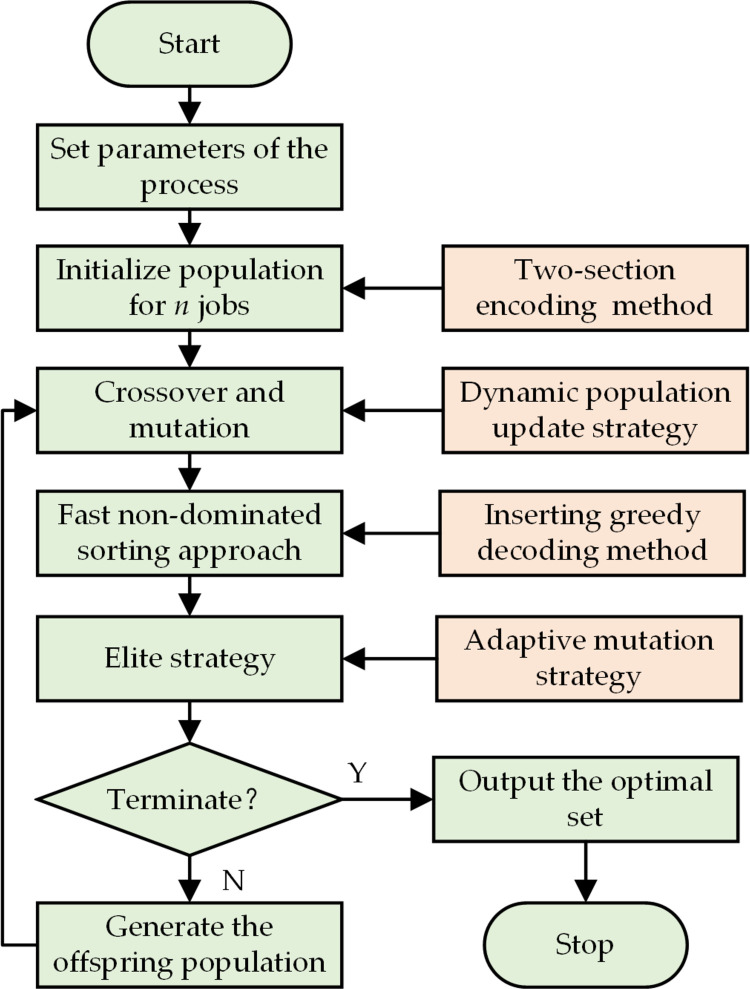
The framework of the proposed NSAG-II method.

The framework of the basic iteration framework is given in Fig 5. A two-section encoding and inserting greedy decoding methods are employed to create initial process planning and scheduling population. The optimal solution set updating strategy was introduced to preserve non-dominated solutions for time, power consumption, and cutting tool consumption, during population updates. The dynamic population update strategy and the adaptive elimination strategy are designed for the elite strategy to enhance both the quality of solutions and population diversity.

The primary steps of the presented method are provided below:

Step 1: Define the parameters for the process planning optimization problem’s working conditions. There are two kinds of parameters. One kind is process parameters such as the machining and processing data in Table 1. The other kind is optimizing parameters for the algorithm including population size, crossover mutation rate, and iteration count.
Step 2: Initialize the population randomly using the two-section encoding method, which is clarified in the Section “Encoding and decoding method”.
Step 3: Crossover and mutation.
Step 4: Decoding all the individuals of the current population, analyze the fitness of each individual of the current population by the fast non-dominated sorting approach, get the best result of the current generation using a non-dominated sorting strategy, and update the optimal set.
Step 5: Update the population and mutation parameters using the elite strategy, which is clarified in the Section “Elite strategy”.
the dynamic population update strategy based on dynamic population update and the adaptive mutation method based on the changing rate of population entropy are selected to enhance both the quality of solutions and population diversity.
Step 6: Verify the algorithm’s condition for termination.
Step 7: Generate the offspring population.
Step 8: Output the optimal resolution.

### Encoding and decoding method

Each individual is made up of two sections. The first represents the operation sequence (OS): the arrangement of the operations assigned to the particular machine. The second represents the machine assignment (MA): the assignment of each operation to a specific machine.

**Fig 6:**
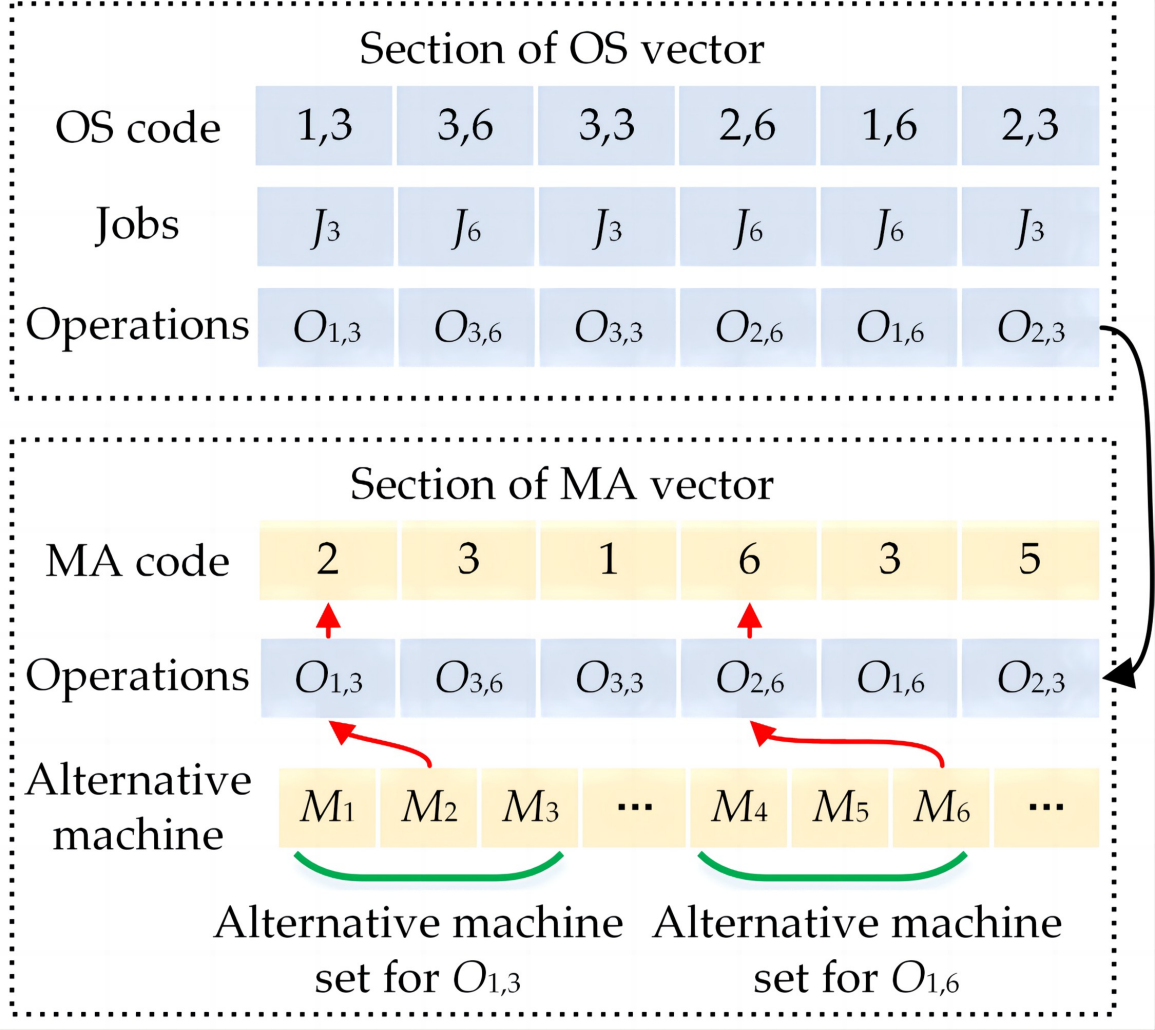
An encoding instance of two jobs.

As shown in Fig 6, assuming that two jobs namely Job 3 and Job 6 (listed in Table 4 in Section 4) are selected for the encoding operation. *O_i,j_* is used to represent the *i* operation of the job *j*. Therefore, a feasible processing order of operations for this chromosome can be:

$$O_{1,3}\to O_{3,6}\to O_{3,3}\to O_{2,6}\to O_{1,6}\to O_{2,3}$$

1. The OS vector: To simplify the MA vector generation process, in this paper, the OS is created in the format of "proces,job". Hence, the OS vector of the chromosome of the above processing order is [1,3 3,6 3,3 2,6 1,6 2,3]. The numbers that make up the OS are the operation indexes: In the OS vector, the operation sequence includes all the jobs, and reflects the machining sequence of all the processes for each job. For instance, the string "1,3" in position one represents Job *J*_3_’s first process; the string “2,6” in position four represents Job *J*_6_’s second process.
2. The MA number reflects the selected machine index. Each number demonstrates a machine number for the related operation number, chosen from the alternative machine set. In Table 4 in Section 4, the alternative machine number of Operation *O*_1,3_ is *M* = {*M*_1_, *M*_2_, *M*_3_}, that is, Machine 1, Machine 2, and Machine 3 are optional for Operation *O*_1,3_ of Job 3. Therefore, according to the alternative machine set for each operation, the MA vector for the OS vector can be [2 3 1 6 3 5]. The length of MA is the same as that of OS, and it represents the full length of machine numbers for all operations of all the jobs.

The decoding process is the procedure of transforming a chromosome code to achievable planning. These are the actions that need to be taken:

Step 1: Read each OS vector for the gene sequence and transform each operation *O*_*i*,*j*_ into the appropriate operation. Read each MA sequence and get the chosen machine number *M*_*k*_ for each operation.
Step 2: Obtain the processing time *pt*_*i*,*j*,*k*_ and loading and unloading time *lt*_*i*,*j*,*k*_ of each operation according to *O*_*i*,*j*,_ and *M*_*k*_.
Step 3: Calculate the power consumption *mc*_*i*,*j*,*k*_, cutting tool consumption *tc*_*i*,*j*,*k*_, and the cost per conversion *mtc*_*i*,*j*,*k*_ according to *O*_*i*,*j*_ and *M*_*k*_.
Step 4: Calculate the total machining time using inserting greedy algorithm. Details of encoding and decoding methods are described in the literature [35].
Step 5: Obtain the total machining cost according to *mc*_*i*,*j*,*k*_, *tc*_*i*,*j*,*k*_, and *mtc*_*i*,*j*,*k*_.

### Population initialization, selection, crossover and mutation

In this paper, the starting OS population and the MA population are generated randomly, as the operation-priority encoding approach does not generate infeasible solutions.

In the chromosome, both the OS and MA sections for the genetic operators are selected by 10% optimal individual retention selection, 50% random selection, and 40% tournament selection.

The crossovers for the OS and MA are carried out independently. The POX crossover [35] is chosen for the OS sequence. The MA sequence is constantly adjusted as the OS sequence changes.

There are three types of mutation: Half random mutation for OS, Full random mutation for OS, and Full random mutation for MA.

1. Half random mutation for OS operation position: Randomly select a chromosome and then one operation *O*_*i*_ of the chromosome. Find the location of the precursor constraint operation *O*_*pr*_ of the same job and the following constraint operation *O*_*po*_ of the same job. *R*andomly select a position *O*_*isrt*_ between these two as the inserting position.
2. Full random mutation for OS operation selection: Randomly select a chromosome and then one operation *O*_*i*_ of the chromosome. Since each feature has alternative operations. Randomly choose an operation out of the optional processing operation collection, and carry out the OS operation selection mutation.
3. Full random mutation for MA machine selection: Randomly select a chromosome and then one operation *O*_*i*_ of the chromosome. Similar to the “OS operation selection”, each operation has alternative machines. Randomly choose a machine from the optional machine collection for operation *O*_*i*_, and carry out the MA mutation.

### Elite strategy

Maintaining the diversity of the optimal set is hampered by the standard NSGA’s ease of local searching area which lead to a local optimal set during the solution phase. Consequently, the dynamic population update strategy and the adaptive mutation method based on the changing rate of population entropy are selected to enhance both the quality of solutions and population diversity.

1. The dynamic population update strategy: For each generation, duplicate chromosomes in the population are deleted and new ones are randomly generated. Hence, the current population is updated by reducing the homogenization of the whole chromosomes.
2. The adaptive mutation method: Δe is used to represent the entropy change rate among two generations. μ is set to represent the threshold value of the entropy change rate of the population. If Δe≥μ, the algorithm runs well. If Δe<μ, mutation probability, and mutation point number are increased to enhance the local search ability. Please refer to the literature [36] for a detailed description.

## Section 4: Real-world case study and discussion

The butterfly valve and the gate valve are the two most commonly used valves in the water flow transportation pipelines of hydroelectric power plants. Almost all the parts are manufactured in the same workshop at the same time. Usually, the process plan is made directly according to the experience of the dispatcher, which leads to low production efficiency. Therefore, the suggested approach is applied to find a better solution for the IPPS problem in the workshop.

### Description of case

**Fig 7:**
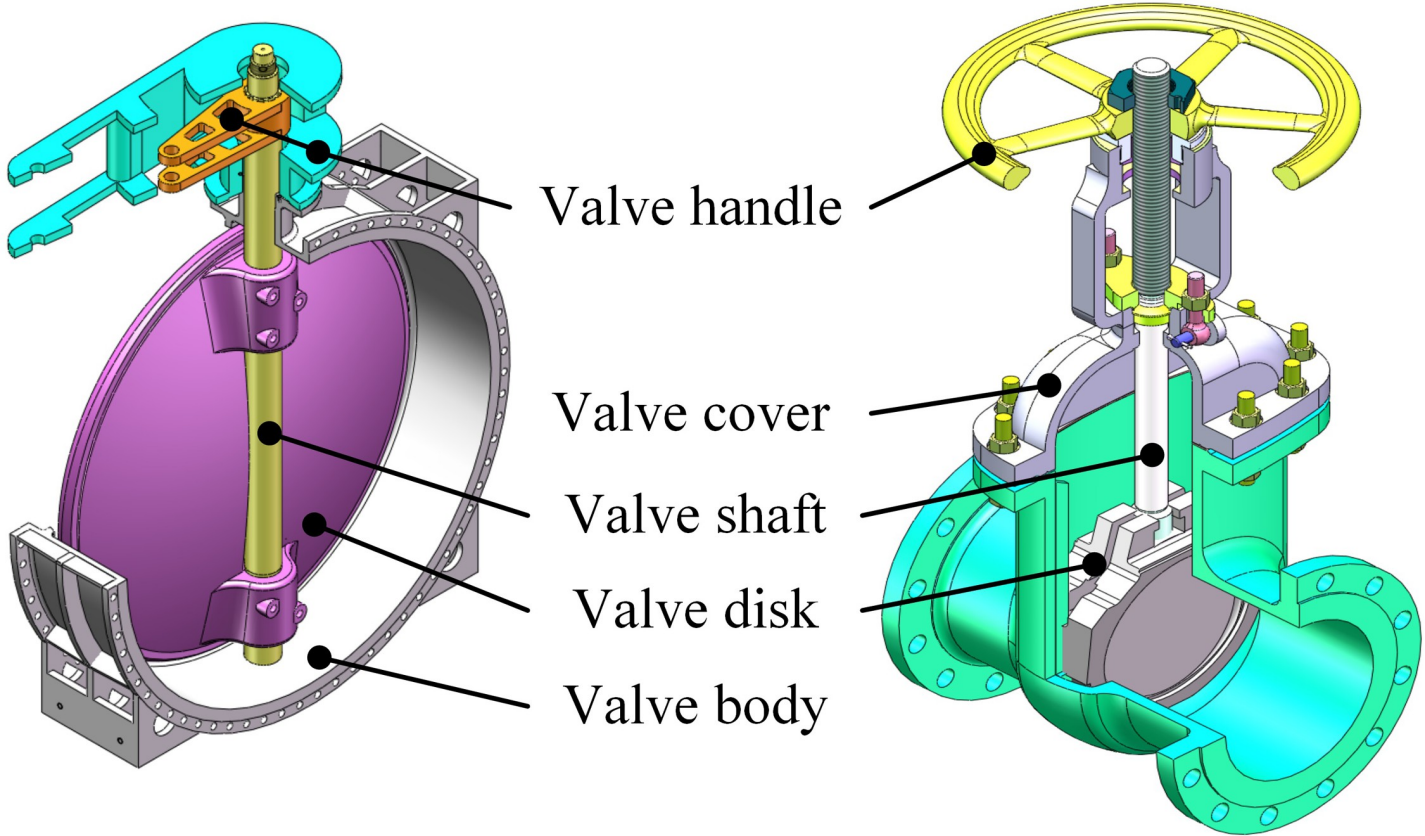
Cross-sectional views of a butterfly valve and a gate valve of large size.

A large valve contains a body, a disk, a shaft, a cover, and one or several handle parts. Fig 7 shows the cross-sectional views of a butterfly valve and a gate valve of Yuanda Valve Company, which is the largest valve manufacturer in China. Take a real purchase order as an example: 9 butterfly valves and 5 gate valves with different models need to be produced in a single order.

### Parameter settings

The valves are machined in the machining workshop shown in Fig 1. The pertinent machine parameters are displayed in Table 3.

**Table 3:** Pertinent parameters of machines.

Parameters of machines	M1	M2	M3	M4	M5	M6	M7	M8
Machine cutting power (KW)	3.3	2.2	1.6	2.3	3.1	4.0	1.2	13.2
Machine idling power (KW)	0.8	0.5	0.3	0.5	0.7	0.9	0.2	0.1
Cutting tool consumption (Pcs/hr)	2.9	2.2	1.7	2.6	3.6	4.8	1.5	0
Loading and unloading time (hr)	0.3	0.2	0.1	0.1	0.4	0.4	0.1	0.1

Except for the valve handle, there are 7 kinds of parts need to be machined in the workshop described in Section 1. The machining process information of all parts is provided in Table 4. The parameters of the proposed NSGA-II algorithm are given in Table 5 for the multi-objective optimization problem of valves.

**Table 4:** Processing information on all the parts for the butterfly valve and the gate valve.

Jobs	Features (description)	Alternative operations	Alternative machines	Machining times (h)	Precedence constraints
Job 1 (butterfly body)	Information is shown in Table 1.				
Job 2 (Butterfly disk)	F1 (Sealing ring surface)	O1/O2	M1,M2,M3/M4,M5,M6	7.2,7.5,8.0/7.5,7.2,6.7	
	F2 (Hole for shaft)	O3	M1,M5,M6	1.5,1.8,1.3	Before F3,F4
	F3 (Groove for shaft)	O4	M1,M5,M6	1.8,2.3,1.5	
	F4 (Thread for shaft)	O5/O6	M1,M5,M6/M7	0.9,1.3,0.6/0.6	
Job 3 (Butterfly shaft)	F1 (OD surface)	O1	M1,M2,M3	6.3,6.0,6.8	First of all
	F2 (Drive slot)	O2	M4,M5,M6	1.2,0.9,0.6	
	F3 (Thread)	O3	M1,M2,M3	0.7,0.9,1.3	
Job 4 (Gate body)	F1 (Flange surfaces for pipe)	O1/O2	M1,M2,M3/M4,M5,M6	5.5,6.0,6.7/5.1,4.5,3.8	Before F2,F5,F6
	F2 (Flange holes for pipe)	O3/O4	M4,M5,M6/M7	1.1,1.5,1.7/1.6	
	F3 (Flange surface for cover)	O5/O6	M1,M2,M3/M4,M5,M6	4.2,4.5,4.8/3.0,2.7,2.4	Before F4
	F4 (Flange holes for cover)	O7/O8	M4,M5,M6/M7	0.8,0.6,0.5/0.4	
	F5 (Inside cylindrical surface)	O9/O10	M1,M2,M3/M4,M5,M6	4.9,5.2,5.5/3.7,3.0,2.9	
	F6 (Inner sealing plane)	O11/O12	M1,M2,M3/M4,M5,M6	1.7,2.2,2.5/1.5,1.2,0.9	Before F7
	F7 (Inner sealing plane)	O13	M8	3.2	
Job 5 (Gate disk)	F1 (Sealing planes)	O1/O2	M1,M2,M3/M4,M5,M6	4.7,5.1,5.5/6.0,5.6,5.3	
	F2 (Pinhole)	O3/O4	M4,M5,M6/M7	3.0,2.7,2.3/3.0	
Job 6 (Gate shaft)	F1 (OD surface)	O1	M1,M2,M3	6.5,6.9,7.4	First of all
	F2 (Drive slot)	O2	M4,M5,M6	1.3,0.9,0.5	
	F3 (Thread)	O3	M1,M2,M3	0.6,0.9,1.3	
Job 7 (Gate cover)	F1 (Flange surface)	O1/O2	M1,M2,M3/M4,M5,M6	3.8,4.3,4.7/4.8,4.5,4.1	Before F2
	F2 (Flange holes)	O3/O4	M4,M5,M6/M7	1.5,1.7,2.2/0.9	
	F3 (Upper surface)	O5/O6	M1,M2,M3/M4,M5,M6	1.7,2.2,2.5/1.8,1.5,1.2	Before F4,F5
	F4 (Hole for shaft)	O7/O8	M1,M2,M3/M4,M5,M6	1.1,1.3,1.5/1.2,1.1,1.0	
	F5 (Circular groove hole)	O9/O10	M1,M2,M3/M4,M5,M6	0.3,0.5,0.7/1.0,0.8,0.6	

**Table 5:** Parameters of the presented method.

Algorithm parameters	Value
Population size	50
Generation count	500
Crossover probability	0.8
The initial value of the mutation probability	0.2
The initial value of the mutation point number	0.5% of the chromosome (Not less than 1 point)
Threshold value of the entropy change rate	0.02
Selection rate	10% by optimal individual retention selection.
	50% by random selection.
	40% by tournament selection.

### Case results and analyses

Following the NSGA-II process flow in Fig 5, the optimization goal is minimizing both the makespan and manufacturing costs which consist of power consumption and cutting tool consumption.

**Fig 8:**
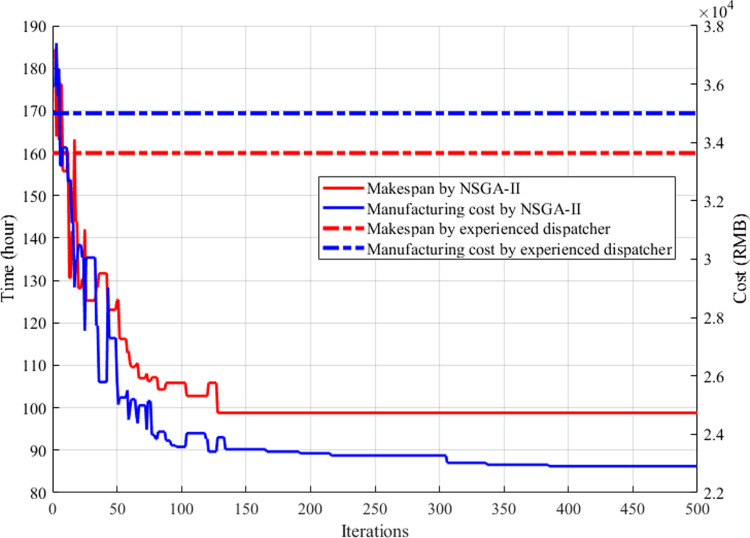
The comparison of two criteria optimization objectives.

The iteration curves of the algorithm are shown in Fig 8, in which the makespan and the manufacturing cost are simultaneously obtained. We can see that the makespan is 98.8 hours and the manufacturing cost is ¥ 22907.8. (1) The time curve starts from the original randomized process planning and scheduling value of 184.2 hours and decreases sharply to 98.8 hours with a decrease of 46.4%. In contrast, the experienced dispatcher’s value (the red dotted line) is about 160 hours. The makespan from the algorithm is about 62% of that from the experience. (2) The cost curve starts at the original random value of ¥ 37406.6 and decreases rapidly to ¥ 22907.8 with a decrease of 38.3%. By contrast, the experienced dispatcher’s value (the blue dotted line) is about ¥ 35000. The cost from the algorithm is about 65% of that from the experience. (3) The time curve and the cost curve follow similar trends. This is because the optimization of operation alignment and machine selection, as the main optimization objectives, contribute to both the shorter makespan and the lower manufacturing cost.

The two curves confirm that the improved NSGA-II algorithm converges quickly during the population search procedure.

**Fig 9:**
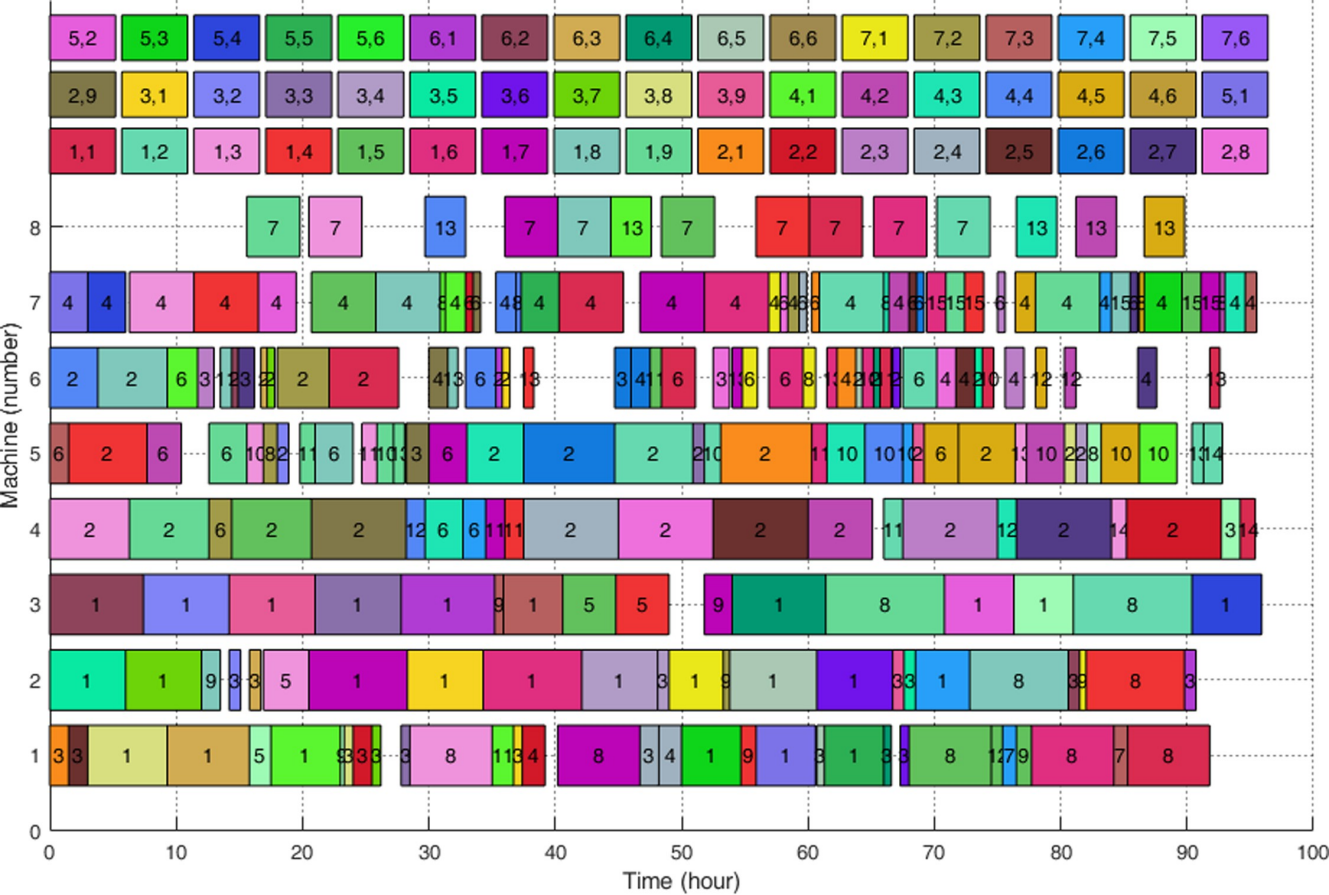
Gantt chart of a best optimal set.

Fig 9 shows the Gantt chart of one of the final optimal sets. It can be observed that the arrangement of processes is compact though blanks exist. It can be concluded that the suggested algorithm can obtain a better result for a valve workshop in the real world and has a high degree of promise for use in practical production scheduling problems.

A detailed process planning plan (a butterfly valve and a gate valve of purchased valves) from a selected solution in an optimal set is displayed in Table 6. The job number is defined as “Job *i*,*j*”, where *i* and *j* represent the valve part number and the purchase order number, separately.

**Table 6:** A detailed process planning arrangement from a chosen solution in an optimal set (partial).

Valve type	Part name	Job No.	Process plans
Butterfly Valve	Body	Job 1,1	O2(M6)-O4(M7)-O6(M6)-O7(M8)-O11(M6)-O10(M6)-O8(M1)-O13(M6)-O14(M4)
	Disk	Job 2,1	O3(M1)-O2(M5)-O6(M7)-O4(M6)
	Shaft	Job 3,1	O1(M3)-O2(M6)-O3(M1)
Gate Valve	Body	Job 4,1	O6(M6)-O1(M1)-O8(M7)-O4(M7)-O11(M1)-O13(M8)-O10(M5)
	Disk	Job 5,1	O4(M7)-O1(M1)
	Shaft	Job 6,1	O1(M3)-O2(M6)-O3(M2)
	Cover	Job 7,1	O1(M2)-O6(M6)-O4(M7)-O8(M6)-O9(M2)

At the same time, several phenomena require attention: (1) The overall utilization of all machines is efficient. The performance of the individual machines was fully utilized and there was no big difference in their working times. (2) Machines 1, 2, and 3 are lathes, and machines 4, 5, and 6 are milling machines. The utilization rate of lathes is higher than that of milling machines in general. The reason should be that large valve parts are mostly rotary body, and turning is more suitable for this type of process. In addition, the rotary section requires a greater amount of cutting. (3) Unlike machines 4 and 5, machine 6 has a relatively low processing time. This is because machine 6 has the highest tool power and tool consumption. Moreover, machine 6 does not have specialized fixtures and the machining efficiency is not proportional to the power. Therefore, it is recommended to upgrade Machine 6, such as adding automatic fixtures to increase processing efficiency, improving the configuration of cutting settings to reduce tool consumption. (4) Machine 8 is the least utilized of all the machines. The reason should be that Machine 8 is a dedicated heat-treating equipment and valve parts require fewer heat-treating processes. It is recommended to share this equipment with other workshops to increase the efficiency of utilization.

## Section 5: Discussion

The aim of this current research is to solve the IPPS problem by using an improved NSGA-II algorithm. In general, this study shows that the proposed algorithm can be extended to real-world case of valve parts. The experimental results also indicate that the algorithm is effective, which is consistent with the existing literature.

The high performance of the improved NSGA-II is mainly due to the following factors. First of all, the encoding and decoding methods in this study enables the algorithm to ensure that the infeasible solutions are seldom generated, and the whole solution space is completely covered. Then, the designed crossover and mutation operators effectively inherit the excellent gene from the parent and help the algorithm to extensively search an area. Finally, the novel elite strategy based on the dynamic population update and the adaptive mutation method makes the search process more effective. At last, experimental results showed that the proposed algorithm is a good principle for the IPPS problem.

## Section 6: Conclusion and future work

In this research, an actual-world IPPS problem from a job shop of a large valve manufacturing company is studied. An improved NSGA-II algorithm is constructed to deal with the problem. A case study from the realistic production shows that the improved algorithm can reduce both makespan and manufacturing costs and that the proposed algorithm is a better method for the IPPS problem in comparison to the schedule of an experienced dispatcher.

The following areas can be the subjects of future research: (1) The efficiency of the presented algorithm could be further improved in the future. A combined algorithm of GA and with other algorithms is worth studying as the next focus. (2) Realistic manufacturing resources are complex, for example, lower utilization of some equipment, and personal preferences of the operator. Hence, the designed mathematical model needs further refinement. (3) Realistic production may run into unpredictable or dynamic events, such as machine failures. Thus, rescheduling and dynamic process planning are required. Hence dynamic process planning and rescheduling is necessary.

## Supporting information

S1 Table Curve data set of the two criteria optimization objectives. (XLSX)

## References

[1] Qian J-y, Gao Z-x, Hou C-w, Jin Z-j. A comprehensive review of cavitation in valves: mechanical heart valves and control valves. Bio-Design and Manufacturing. 2019;2(2):119–36.

[2] Xia W, Wu Z. An effective hybrid optimization approach for multi-objective flexible job-shop scheduling problems. Computers & industrial engineering. 2005;48(2):409–25.

[3] Sugimura N, Hino R, Moriwaki T. Integrated process planning and scheduling in holonic manufacturing systems. Proceedings of the 2001 IEEE International Symposium on Assembly and Task Planning (ISATP2001) Assembly and Disassembly in the Twenty-first Century(Cat No 01TH8560); 2001: IEEE.

[4] Wang L, Shen W. Process planning and scheduling for distributed manufacturing: Springer Science & Business Media; 2007.

[5] Wang Y, Zhang Y, Fuh JY, Zhou Z, Xue L, Lou P, editors. A web-based integrated process planning and scheduling system. 2008 IEEE International Conference on Automation Science and Engineering; 2008: IEEE.

[6] Xia H, Li X, Gao L. A hybrid genetic algorithm with variable neighborhood search for dynamic integrated process planning and scheduling. Computers & Industrial Engineering. 2016;102:99–112.

[7] Goli A, Tirkolaee E B. Designing a portfolio-based closed-loop supply chain network for dairy products with a financial approach: accelerated benders decomposition algorithm. Computers & Operations Research, 2023, 155: 106244.

[8] Montazerolghaem A. Optimized software-defined multimedia framework: networking and computing resource management. Journal of Ambient Intelligence and Humanized Computing, 2023, 14(9): 12981–13001.

[9] Goli A. Integration of blockchain-enabled closed-loop supply chain and robust product portfolio design. Computers & Industrial Engineering, 2023, 179: 109211.

[10] Alhilali A H, Montazerolghaem A. Artificial intelligence based load balancing in SDN: a comprehensive survey. Internet of Things, 2023: 100814.

[11] Li X, Zhang C, Gao L, Li W, Shao X. An agent-based approach for integrated process planning and scheduling. Expert Systems with Applications. 2010;37(2):1256–64.

[12] Mohammadi G, Karampourhaghghi A, Samaei F. A multi-objective optimisation model to integrating flexible process planning and scheduling based on hybrid multi-objective simulated annealing. International Journal of Production Research. 2012;50(18):5063–76.

[13] Li X, Gao L, Wang W, Wang C, Wen L. Particle swarm optimization hybridized with genetic algorithm for uncertain integrated process planning and scheduling with interval processing time. Computers & Industrial Engineering. 2019;135:1036–46.

[14] Davis L. Job shop scheduling with genetic algorithms. Proceedings of the first International Conference on Genetic Algorithms and their Applications; 2014: Psychology Press.

[15] Shokouhi E. Integrated multi-objective process planning and flexible job shop scheduling considering precedence constraints. Production & Manufacturing Research. 2018;6(1):61–89.

[16] Zhang L, Wong T. An object-coding genetic algorithm for integrated process planning and scheduling. European Journal of Operational Research. 2015;244(2):434–44.

[17] Mohapatra P, Nayak A, Kumar S, Tiwari M. Multi-objective process planning and scheduling using controlled elitist non-dominated sorting genetic algorithm. International journal of production research. 2015;53(6):1712–35.

[18] Luo G, Wen X, Li H, Ming W, Xie G. An effective multi-objective genetic algorithm based on immune principle and external archive for multi-objective integrated process planning and scheduling. The International Journal of Advanced Manufacturing Technology. 2017;91:3145–58.

[19] Amin-Naseri M, Afshari AJ. A hybrid genetic algorithm for integrated process planning and scheduling problem with precedence constraints. The International Journal of Advanced Manufacturing Technology. 2012;59:273–87.

[20] Sun K, Zheng D, Song H, Cheng Z, Lang X, Yuan W, et al. Hybrid genetic algorithm with variable neighborhood search for flexible job shop scheduling problem in a machining system. Expert Systems with Applications. 2023;215:119359.

[21] Verma S, Pant M, Snasel V. A comprehensive review on NSGA-II for multi-objective combinatorial optimization problems. IEEE Access, 2021, 9: 57757–57791.

[22] Mohapatra P, Benyoucef L, Tiwari M. Integration of process planning and scheduling through adaptive setup planning: a multi-objective approach. International Journal of Production Research. 2013;51(23–24):7190–208.

[23] Guo K, Liang Y, Niu M, Tan W. Integrated optimization of process planning and scheduling problems based on complex networks. Journal of Industrial Information Integration. 2023;36:100533.

[24] Wen X, Wang K, Li H, Sun H, Wang H, Jin L. A two-stage solution method based on NSGA-II for green multi-objective integrated process planning and scheduling in a battery packaging machinery workshop. Swarm and Evolutionary Computation. 2021;61:100820.

[25] Qiong L, Zhen M. Integrated optimization of process planning and shop scheduling for reducing manufacturing carbon emissions. Journal of Mechanical Engineering. 2017;53(11):164–74.

[26] Zhang C, Gu P, Jiang P. Low-carbon scheduling and estimating for a flexible job shop based on carbon footprint and carbon efficiency of multi-job processing. Proceedings of the Institution of Mechanical Engineers, Part B: Journal of Engineering Manufacture. 2015;229(2):328–42.

[27] Deng Q, Gong G, Gong X, Zhang L, Liu W, Ren Q. A bee evolutionary guiding nondominated sorting genetic algorithm II for multiobjective flexible job-shop scheduling. Computational Intelligence and Neuroscience. 2017;2017:5232518. doi: 10.1155/2017/5232518 . 28458687 PMC5387816

[28] Wang X, Wang B, Zhang X, Xia X, Pan Q. Two-objective robust job-shop scheduling with two problem-specific neighborhood structures. Swarm and Evolutionary Computation. 2021;61:100805.

[29] Wen X, Qian Y, Lian X, Zhang Y, Wang H, Li H. Improved genetic algorithm based on multi-layer encoding approach for integrated process planning and scheduling problem. Robotics and Computer-Integrated Manufacturing, 2023, 84: 102593.

[30] Liu Q, Li X, Gao L. A modified genetic algorithm with new encoding and decoding methods for integrated process planning and scheduling problem. IEEE Transactions on Cybernetics, 2020, 51(9): 4429–4438. 10.1109/TCYB.2020.3026651 33055056

[31] Fan J, Zhang C, Liu Q, Shen W, Gao L. An improved genetic algorithm for flexible job shop scheduling problem considering reconfigurable machine tools with limited auxiliary modules. Journal of Manufacturing Systems. 2022;62:650–67.

[32] Zhang X, Liao Z, Ma L, Yoa J. Hierarchical multistrategy genetic algorithm for integrated process planning and scheduling. Journal of Intelligent Manufacturing, 2022, 33(1): 223–246.

[33] Wen X, Song Q, Qian Y, Qiao D, Wang H, Zhang Y, et al. Effective improved NSGA-II algorithm for multi-objective integrated process planning and scheduling. Mathematics. 2023;11(16):3523.

[34] Kim YK, Park K, Ko J. A symbiotic evolutionary algorithm for the integration of process planning and job shop scheduling. Computers & operations research. 2003;30(8):1151–71.

[35] Gu X, Huang M, Liang X. An improved genetic algorithm with adaptive variable neighborhood search for FJSP. Algorithms. 2019;12(11):243.

[36] Dinghui W, Hongcai O, Yong Z, Jing W. Multi-cycle steel energy planning optimization based on improved NSGA-II algorithm. 2022 34th Chinese Control and Decision Conference (CCDC); 2022: IEEE.

